# Effect of touch on proprioception: non-invasive trigeminal nerve stimulation suggests general arousal rather than tactile-proprioceptive integration

**DOI:** 10.3389/fnhum.2024.1429843

**Published:** 2024-10-14

**Authors:** Justin Tanner, Gerrit Orthlieb, Stephen Helms Tillery

**Affiliations:** School of Biological and Health Systems Engineering, Arizona State University, Tempe, AZ, United States

**Keywords:** proprioception, trigeminal nerve stimulation, cranial nerve stimulation, tactile-proprioceptive integration, neuromodulation

## Abstract

**Introduction:**

Proprioceptive error of estimated fingertip position in two-dimensional space is reduced with the addition of tactile stimulation applied at the fingertip. Tactile input does not disrupt the participants’ estimation strategy, as the individual error vector maps maintain their overall structure. This relationship suggests integration of proprioception and tactile information improves proprioceptive estimation, which can also be improved with trained mental focus and attention. Task attention and arousal are physiologically regulated by the reticular activating system (RAS), a brainstem circuit including the locus coeruleus (LC). There is direct and indirect evidence that these structures can be modulated by non-invasive trigeminal nerve stimulation (nTNS), providing an opportunity to examine nTNS effect on the integrative relationship of proprioceptive and tactile information.

**Methods:**

Fifteen right-handed participants performed a simple right-handed proprioceptive estimation task with tactile feedback (touch) and no tactile (hover) feedback. Participants repeated the task after nTNS administration. Stimulation was delivered for 10 min, and stimulation parameters were 3,000 Hz, 50 μs pulse width, with a mean of 7 mA. Error maps across the workspace are generated using polynomial models of the participants’ target responses.

**Results:**

Error maps did not demonstrate significant vector direction changes between conditions for any participant, indicating that nTNS does not disrupt spatial proprioception estimation strategies. A linear mixed model regression with nTNS epoch, tactile condition, and the interaction as factors demonstrated that nTNS reduced proprioceptive error under the hover condition only.

**Discussion:**

We argue that nTNS does not disrupt spatial proprioceptive error maps but can improve proprioceptive estimation in the absence of tactile feedback. However, we observe no evidence that nTNS enhances tactile-proprioceptive integration as the touch condition does not exhibit significantly reduced error after nTNS.

## 1 Introduction

Non-invasive electrical neuromodulation provides an accessible, chemical free method to successfully induce systemic and effective changes in the cortex with short latency. Trigeminal and vagus nerve stimulation have seen recent success, altering brain state and helping treat anxiety (George et al., 2008; Hartley et al., 2023; Xiao et al., 2020; Burger et al., 2019; Verkuil and Burger, 2019), depression (Liu et al., 2020; Fang et al., 2016; Liu et al., 2018; Trevizol and Cordeiro, 2017), PTSD (Koek et al., 2019; Bottari et al., 2024; Trevizol et al., 2016), headache (Alt et al., 2020; Vecchio et al., 2018; Silberstein et al., 2016; Boström et al., 2019), and epilepsy (Xue et al., 2022; Hödl et al., 2020; Pop et al., 2011). Success has been seen in invasive implanted devices as well as non-invasive transcutaneous devices. The benefit of non-invasive trigeminal nerve stimulation (nTNS) and non-invasive vagus nerve stimulation (nVNS) exists in the accessibility of the method, requiring no implants nor surgery, and demonstrated effective somatosensory modulation.

The vagus and trigeminal nerves project directly to the brainstem (van Boekholdt et al., 2020; de Cicco et al., 2018; Broncel et al., 2020; Luckey et al., 2023; Schwarz and Luo, 2015; Zerari-Mailly et al., 2005), providing an avenue to affect cortical circuits regulating autonomic activity, including the reticular activating system (RAS) with the locus coeruleus (LC). The RAS and LC regulate arousal, attention, sleep, and somatosensory behaviors (Khroud et al., 2020). Multiple approaches demonstrate evidence that cranial nerve stimulation modulates these brainstem nuclei and consequentially modulates neocortex activity. Using blink reflex as an indirect model of subcortical activity, nTNS is shown to modulate brainstem circuits with long-term effects (Mercante et al., 2015; Pilurzi et al., 2016). In rodent models, cranial nerve stimulation directly drives LC firing rate (Hulsey et al., 2017), and increases cFOS expression in RAS nuclei and somatosensory cortex—indicating increased neural activity (Mercante et al., 2017). In humans, nTNS suppresses proprioceptive brainstem nuclei and nVNS activates the nucleus of the solitary tract and the LC, both integral to the RAS (Yakunina et al., 2017).

There is strong evidence of successful modulation of somatosensory perception and behavior via cranial nerve stimulation. Closed loop cranial nerve stimulation, specifically vagus nerve stimulation, has been successful in neurorehabilitation of motor function and tactile sensitivity after nerve damage or stroke (Darrow et al., 2020; Kilgard et al., 2018; Meyers et al., 2019). In these cases, neuromodulation was able to decrease pain sensitivity (Busch et al., 2013; Lerman et al., 2019), increase pain and pressure thresholds (Kilgard et al., 2018), and modulate tactile sensitivity (Darrow et al., 2020). Investigating nTNS in psychophysical tasks, it can improve motor learning (Arias and Buneo, 2022) or can decrease vigilance and attention depending on stimulation parameters (Piquet et al., 2011).

In summary, the neuroanatomical pathways linking both vagus and trigeminal nerves to relevant brainstem nuclei are similar. Benefits in anxiety, depression, and other pathologies are consistently observed with both modalities. Somatosensory plasticity and even changes in tactile-proprioceptive integration can be observed after neurostimulation as well. Reaction time tasks, measuring alertness and arousal, consistently demonstrate improvement in participants who are healthy (Lerman et al., 2022; Jongkees et al., 2018), enduring sleep deprivation (McIntire et al., 2021), or diagnoses with attention-deficit/hyperactivity disorder (McGough et al., 2019) that is linked to EEG alpha oscillations (Chen et al., 2023). We aim to further demonstrate the effect of nTNS on somatosensory and proprioceptive integration in an established task (Rincon-Gonzalez et al., 2011; Tanner et al., 2021) that requires a cognitive response evaluating perception rather than an reaction-time task relying on instinctual responses.

## 2 Methods

*Participants*. For this experiment, 17 participants were recruited to perform a right-handed proprioceptive estimation task with non-invasive trigeminal nerve stimulation. The task, parameters, and experimental protocols were reviewed and approved by the Institutional Review Board at Arizona State University.

*Handedness*. Handedness was self-reported by each participant, and the Edinburgh Handedness Inventory questionnaire (Oldfield, 1971) was used to confirm the handedness of each participant before the experiment. Only right-handed participants were included in the subsequent analysis as we have found differences in performance between the dominant and non-dominant hands (Rincon-Gonzalez et al., 2011), especially under novel stimulus conditions (Tanner et al., 2021). Out of the 17 participants recruited, 15 were right-handed with a mean age of 21.8 years old.

**Figure 1:**
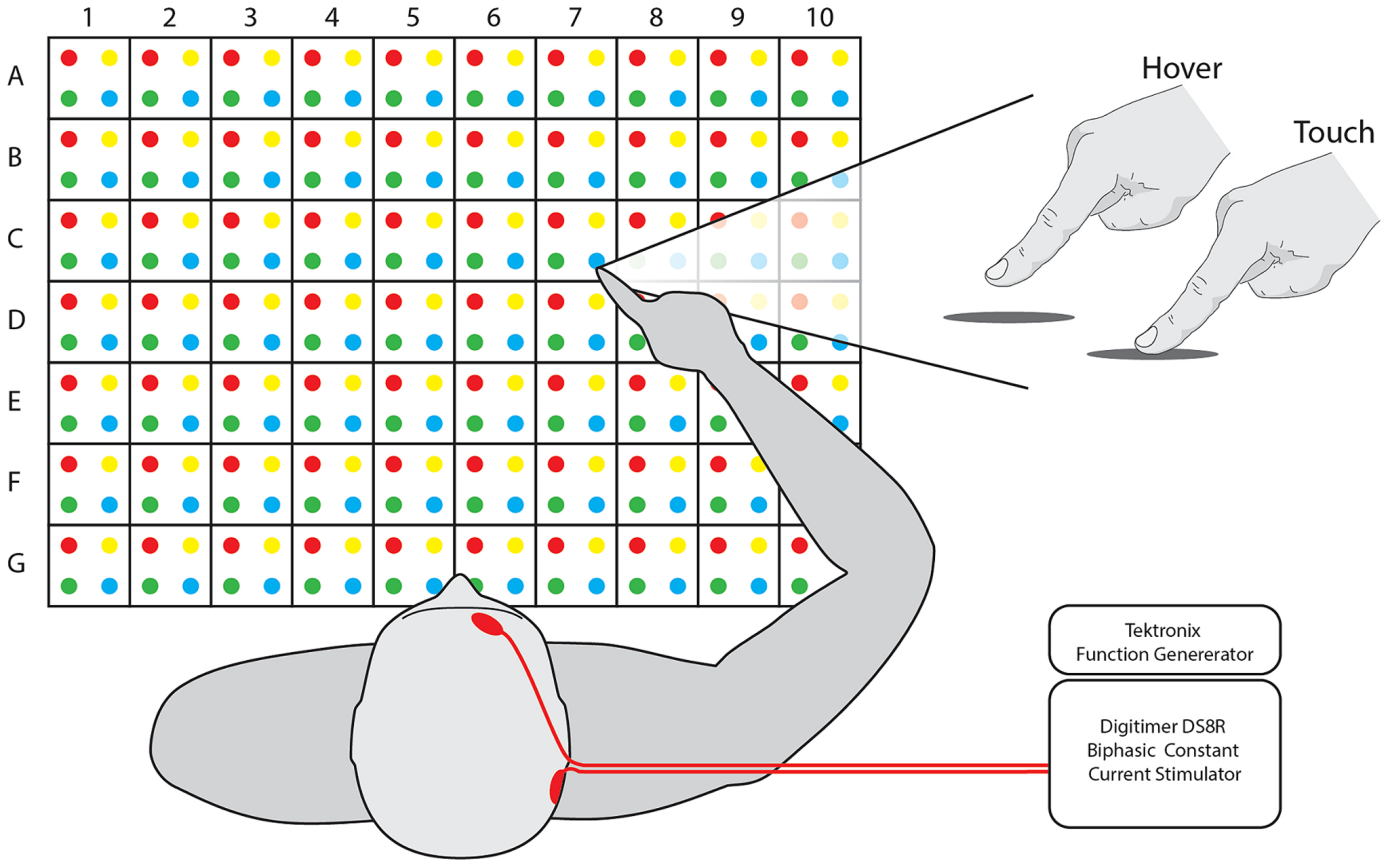
Workspace and feedback. [Left] Participants sit in front of a grid of dots with 2.5 cm spacing between them, creating a workspace 50 cm wide and 35 cm deep. Targets and responses are referred to via their numerical value, alphabetical letter, and color, e.g., A1Red. [Right] Participant finger position for each feedback mode. [Inset] nTNS was delivered with Tektronix and Digitimer equipment. Electrode placement is indicated on the illustrated participant’s head.

*Task*. Participants sat in front of a table with a 50 cm wide and 35 cm deep grid, consisting of 280 targets with alphanumeric and color assignments (Figure 1). A set of 75 random targets was chosen for each participant. Target sets were consistent for all iterations of a participant session and randomized between blocks. For each trial of the task, the participants held their hand a few centimeters above the edge of the workspace midline, close to their chest. With the participant’s eyes closed, the experimenter guided the participant’s hand to a target over the grid, held the hand in position with or without other input for 5 s, and then guided the hand back to the starting position. The participant could then open their eyes and, without moving their arm, report the estimated target by alphanumeric value and color, e.g., “A1Red.” At least one practice trial was explained and administered followed by verbal confirmation of trial comprehension. Trials were only aborted and repeated if the experimenter accidentally touched the participants hand to the table in the process of approaching the target.

The task was performed in four separate blocks: for both hover and touch conditions before and after administration of nTNS. This resulted in four blocks of two factors with two levels: Condition [Hover and Touch] and Epoch [Baseline and nTNS]. In the *Hover* condition, the participant’s hand was kept above the table and no tactile input was provided before returning to the starting position. In the *Touch* condition, the experimenter moved the participant’s hand to the target, then vertically lowered it to the table, allowing contact for 5 s before raising the hand and returning it to the starting position.

Neuromodulatory stimulation was delivered via a Digitimer DS8R stimulator after the baseline blocks of hover and touch trials. The positive electrode was placed above the right eyebrow, near the foramen of the ophthalmic branch of the trigeminal nerve, and the ground was placed on the mastoid behind the ipsilateral ear. We used 1.5″ round Axelgaard PALS neurostimulation electrodes and adhered them in place with medical tape if necessary. The stimulation was delivered as biphasic, symmetrical 50 microsecond pulses delivered at 3,000 Hz (Arias and Buneco, 2022). Pulse amplitude was determined individually for each participant, starting at 2 mA and increasing until the participant reported the sensation as “tolerable but clear,” (4–9 mA, mean = 7 mA). The stimuli were controlled with a Tektronix function generator set to stimulate in one of two timing paradigms. The “Constant” paradigm consists of constant stimulation over a 10-min window. The “Cycling” paradigm consists of stimulation cycled on and off at 30 s intervals across a 10-min window. Participants were randomly separated into one paradigm group, resulting in 8 participants for Constant and 7 participants for Cycle. Immediately following the nTNS delivery, the participants performed the second set of hover and touch trials. Due to low sample size and to ensure statistical confidence, no comparisons between nTNS paradigms were analyzed. All 15 participants were pooled into a single group and considered equal as recipients of nTNS.

**Figure 2:**
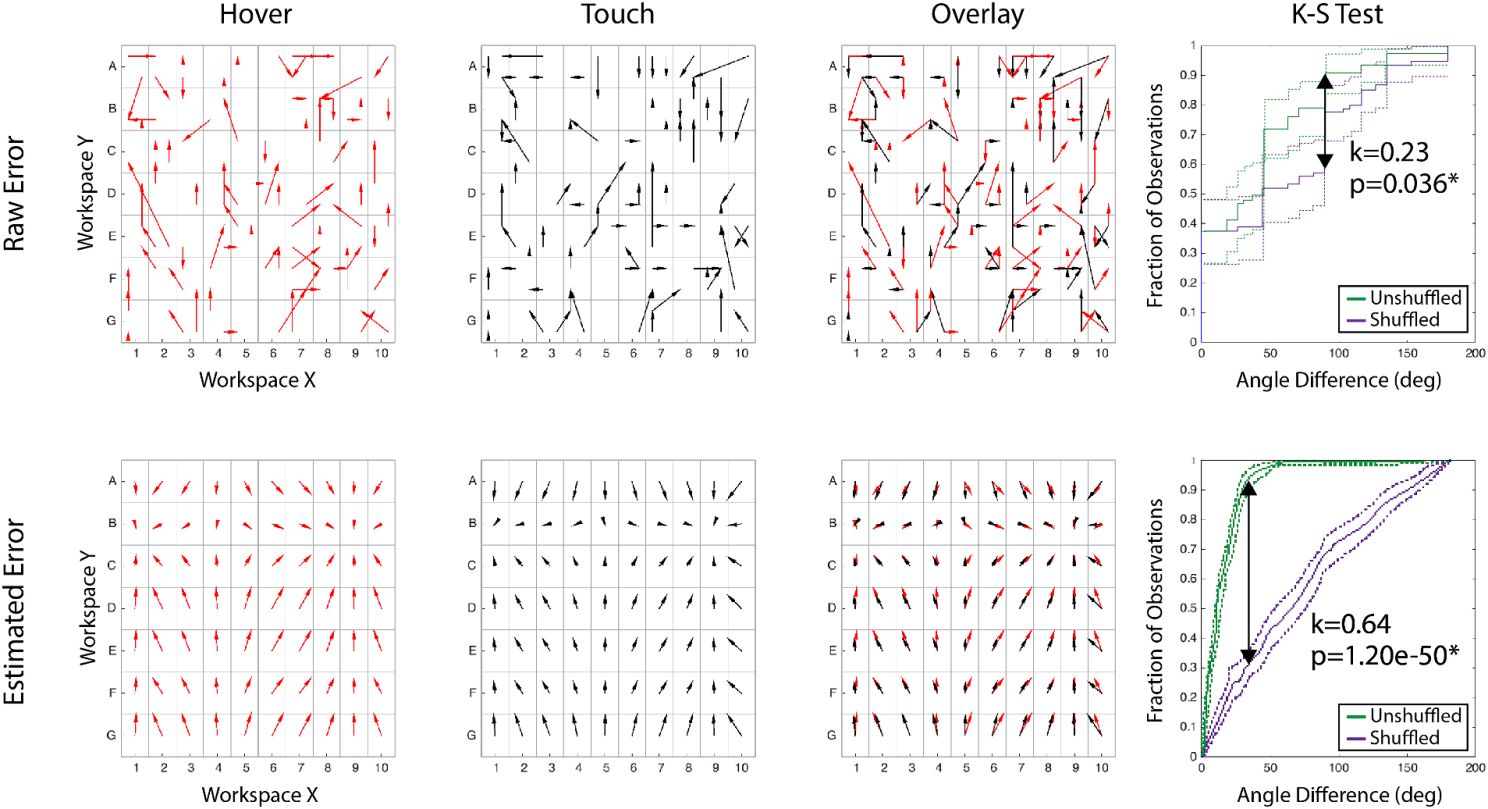
Data processing example. Arrows show proprioceptive error magnitude and direction for the hover and touch conditions. Each tail is the target, and each head is the response. [Top Row] Raw error of actual responses to targets for two conditions, the overlay, and the comparison of the error maps. [Bottom Row] Modeled error of the same conditions constructed for each alphanumeric grid. The right column demonstrates the K-S test statistical process: the maximum span between the shuffled and unshuffled error distributions is denoted as ‘k’ and accompanied by the *p*-value of the test.

*Analysis*. Using a participant’s estimations of the 75 targets, we first transformed the raw error magnitude and direction across the entire sampled workspace. To obtain estimations across the entire workspace, X and Y components of the error vector were modeled with 4th order polynomial regressions. We used these models to calculate an error estimate for each of the 280 potential targets. It is necessary to this analysis that the targets are consistent across each block in order compare the results across sessions. Figure 2 illustrates this process for a single participant’s raw and calculated error from feedback conditions before stimulation. The first two columns illustrate the error maps alone, but the third column overlays the two maps to visualize comparison. Both error map shape and error magnitude need to be evaluated statistically, and all reported analyses were performed on each the calculated error generated from the polynomial models (bottom row of Figure 2).

To evaluate the individual difference of error magnitude between blocks, a Wilcoxon sign-ranked test was performed on the vector magnitudes. This was performed within participants for each pair of blocks, and Bonferroni corrected for multiple comparisons.

For a comprehensive analysis of the effect nTNS has on proprioceptive error magnitude, a linear mixed-effect model (Equation 1) was implemented with Epoch and Condition as factors, including the interaction effect. For the independent variable, we used proprioceptive error for each potential target. Participant ID was included as a random effect to mitigate discrepancies in baseline performance.


(1) $$\begin{array}{c} \mathrm{Estimated}\;{\mathrm{Error}}_{ij}=\beta_{0}+\beta_{1}{\mathrm{Epoch}}_{ij}+\beta_{2}{\mathrm{Condition}}_{ij}\\ +\beta_{3}{\left(\mathrm{Epoch}\times \mathrm{Condition}\right)}_{ij}+u_{0j}+\epsilon_{ij} \end{array}$$

To determine if the shape of the error map is maintained between blocks, we employed the Kolmogorov–Smirnov (K-S) test. Thoroughly explained in Rincon-Gonzalez et al. (2011), the K-S test non-parametrically compares the distribution of two variables. Comparing error distributions directly between blocks does not adequately account for variance across the workspace. In this study, we use the K-S test to compare the difference in error distribution (angle between response vectors of two blocks) and a randomized error distribution (angle between response vector of two blocks, with the second block’s vectors shuffled across the workspace). An unshuffled distribution represents each target’s error vector’s angular difference between feedback modes. A shuffled distribution is built by finding the angular difference between one feedback mode’s actual error vector at a target and a randomly shuffled target’s error vector for the second mode. If the unshuffled error vectors are similar, the angular difference is often small, creating a steep cumulative distribution function (CDF). Both an error map with varied vector differences and a shuffled set would have CDFs that are more linear. Therefore, if the maximum difference between of the populations’ CDFs is sufficiently large, then the error map shapes are significantly similar. An example of this is presented in the far-right column of Figure 2 for both raw and calculated error, displaying the shuffled and unshuffled error distributions. As proprioceptive error maps are idiosyncratic, K-S tests were performed within participants for each pair of blocks, and Bonferroni corrected for multiple comparisons.

## 3 Results

To evaluate the effect of nTNS on the map of proprioceptive error, the magnitude and shape of error were tested between blocks: hover and touch before and after nTNS. These experiments provided raw error across 75 targets of the workspace (Figure 2, Top Row). Analyses were then performed on 4th order polynomial models of each participants’ X and Y error, evaluated at each of the 280 target locations (Figure 2, Bottom Row). R^2^ coefficients of the 4th order polynomial fits were at minimum 0.92, averaged 0.96, and are displayed in Table 1. Using the resultant vectors, a Kolmogorov–Smirnov test compared the spatial structure between modes and a two-sample t-test compared the magnitude of error.

**Table 1:** Regression coefficients for polynomial fits.

			Baseline		nTNS	
nTNS mode			Hover	Touch	Hover	Touch
Cycled	SR	X	0.92	0.96	0.96	0.96
		Y	0.95	0.94	0.95	0.96
	AN	X	0.96	0.98	0.97	0.97
		Y	0.98	0.97	0.97	0.97
	CM	X	0.96	0.98	0.98	0.97
		Y	0.96	0.97	0.97	0.96
	JA	X	0.98	0.98	0.98	0.98
		Y	0.97	0.98	0.97	0.97
	EH	X	0.98	0.98	0.97	0.97
		Y	0.98	0.98	0.97	0.98
	CC	X	0.98	0.99	0.99	0.98
		Y	0.98	0.98	0.98	0.98
	DR	X	0.98	0.96	0.95	0.95
		Y	0.95	0.95	0.95	0.96
Constant	RN	X	0.97	0.97	0.95	0.97
		Y	0.94	0.98	0.97	0.98
	SD	X	0.97	0.98	0.97	0.97
		Y	0.98	0.98	0.98	0.98
	NB	X	0.97	0.96	0.94	0.97
		Y	0.97	0.97	0.97	0.97
	JM	X	0.95	0.97	0.97	0.98
		Y	0.97	0.98	0.97	0.98
	MH	X	0.95	0.96	0.96	0.97
		Y	0.97	0.96	0.93	0.94
	NJ	X	0.96	0.99	0.96	0.98
		Y	0.96	0.97	0.97	0.95
	TL	X	0.96	0.96	0.95	0.94
		Y	0.95	0.96	0.97	0.96

Modeled error for X and Y components of raw error vectors (target to response) for each participant in both conditions of both epochs.

**Figure 3:**
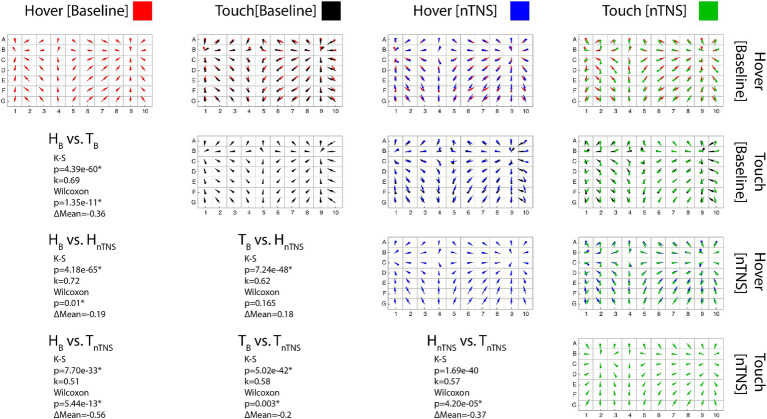
Error maps comparisons. [Diagonal] Each error map shows the proprioceptive error direction and magnitude across the workspace for a single participant. (Red: Hover at Baseline; Black: Touch at Baseline; Blue: Hover after Modulation; Green: Touch after Modulation) [Upper Triangle] Conditions overlayed in each row/column pair to compare maps. [Lower Triangular] Statistical results for each pair. K-S significance implies the maps possess statistically similar shapes. Wilcoxon significance implies a difference in the error means, where positive ΔM values indicate increased mean error in the latter mode.

Error maps of each block and comparisons between each block for a single participant, as well as that participant’s statistical results, are included in Figure 3. Visually, it is apparent the spatial structure is maintained across all modes and as detailed in Table 2; the significant K-S test results corroborate. Table 2 outlines the statistical results of both tests with respective k values (spatial structure) and change in mean error (ΔM). Comparing vector directions using the K-S test shows significantly similar spatial structures in 90/90 cases. Neither tactile condition nor nTNS epoch alters the spatial structure of individual proprioceptive error in this task.

**Table 2:** Statistical results between conditions.

nTNS Mode		Hover [Baseline] and Touch [Baseline]	Hover [Baseline] and Hover [nTNS]	Hover [Baseline] and Touch [nTNS]	Touch [Baseline] and Hover [nTNS]	Touch [Baseline] and Touch [nTNS]	Hover [nTNS] and Touch [nTNS]
Cycled	SR	ΔM = −0.86 cm*	ΔM = 0.92 cm*	ΔM = −0.1 cm	ΔM = 1.78 cm*	ΔM = 0.76 cm*	ΔM = −1.02 cm*
		k = 0.32*	k = 0.32*	k = 0.28*	k = 0.57*	k = 0.41*	k = 0.69*
	AN	ΔM = −0.98 cm*	ΔM = −1.52 cm*	ΔM = −1.13 cm*	ΔM = −0.54 cm*	ΔM = −0.15 cm	ΔM = 0.39 cm*
		k = 0.65*	k = 0.52*	k = 0.55*	k = 0.61*	k = 0.55*	k = 0.49*
	CM	*ΔM = −0.31 cm* ^†^	ΔM = 0.06 cm	ΔM = −0.49 cm*	ΔM = 0.37 cm*	ΔM = −0.17 cm	ΔM = −0.55 cm*
		k = 0.46*	k = 0.39*	k = 0.41*	k = 0.65*	k = 0.49*	k = 0.7*
	JA	ΔM = 0.49 cm*	ΔM = 0.18 cm	ΔM = 1.37 cm*	ΔM = −0.31 cm*	ΔM = 0.88 cm*	ΔM = 1.19 cm*
		k = 0.22*	k = 0.44*	k = 0.27*	k = 0.32*	k = 0.19*	k = 0.38*
	EH	ΔM = −1.39 cm*	ΔM = −1.09 cm*	ΔM = −1.46 cm*	ΔM = 0.3 cm	ΔM = −0.07 cm	*ΔM = −0.38 cm* ^†^
		k = 0.27*	k = 0.25*	k = 0.22*	k = 0.51*	k = 0.57*	k = 0.38*
	CC	ΔM = 0.08 cm^†^	ΔM = −0.62 cm*	ΔM = −0.9 cm*	ΔM = −0.7 cm*	ΔM = −0.98 cm*	ΔM = −0.28 cm*
		k = 0.65*	k = 0.71*	k = 0.55*	k = 0.6*	k = 0.49*	k = 0.56*
	DR	ΔM = −0.73 cm*	ΔM = −1.16 cm*	ΔM = −0.24 cm	ΔM = −0.42 cm*	ΔM = 0.49 cm*	ΔM = 0.91 cm*
		k = 0.5*	k = 0.46*	k = 0.6*	k = 0.48*	k = 0.53*	k = 0.44*
Constant	RN	ΔM = −1.08 cm*	ΔM = −1.05 cm*	ΔM = −1.49 cm*	ΔM = 0.04 cm	ΔM = −0.41 cm*	ΔM = −0.44 cm*
		k = 0.17*	k = 0.23 *	k = 0.31*	k = 0.35*	k = 0.29*	k = 0.42*
	SD	ΔM = −0.36 cm*	*ΔM = −0.19 cm* ^†^	ΔM = −0.56 cm*	ΔM = 0.18 cm	ΔM = −0.2 cm*	ΔM = −0.37 cm*
		k = 0.71*	k = 0.71*	k = 0.59*	k = 0.6*	k = 0.46*	k = 0.57*
	NB	ΔM = 0.93 cm*	ΔM = 1.27 cm*	*ΔM = 0.61 cm* ^†^	ΔM = 0.34 cm	*ΔM = −0.32 cm* ^†^	*ΔM = −0.66 cm* ^†^
		k = 0.56*	k = 0.5*	k = 0.44*	k = 0.77*	k = 0.62*	k = 0.67*
	JM	ΔM = −0.55 cm*	ΔM = −0.18 cm	ΔM = −1.1 cm*	*ΔM = 0.37 cm (p = 0.01)* ^†^	ΔM = −0.55 cm*	ΔM = −0.92 cm*
		k = 0.39 *	k = 0.32*	k = 0.48*	k = 0.44*	k = 0.31*	k = 0.26*
	MH	*ΔM = 0.35 cm* ^†^	ΔM = −0.24 cm	ΔM = −0.56 cm*	ΔM = −0.59 cm*	ΔM = −0.9 cm*	ΔM = −0.32 cm*
		k = 0.61*	k = 0.43*	k = 0.69*	k = 0.29*	k = 0.62*	k = 0.37*
	NJ	ΔM = −1.56 cm*	ΔM = −1.15 cm*	ΔM = −0.79 cm*	ΔM = 0.41 cm*	ΔM = 0.78 cm*	ΔM = 0.36 cm*
		k = 0.27*	k = 0.34*	k = 0.33*	k = 0.29*	k = 0.35*	k = 0.47*
	TL	ΔM = −0.11 cm	ΔM = 0.17 cm*	ΔM = 0.04 cm	ΔM = 0.28 cm*	ΔM = 0.15 cm	*ΔM = −0.13 cm* ^†^
		k = 0.66*	k = 0.44*	k = 0.44*	k = 0.42*	k = 0.36*	k = 0.7*
	TN	ΔM = −0.21 cm*	ΔM = −0.63 cm*	*ΔM = 0.11 cm* ^†^	ΔM = −0.42 cm*	ΔM = 0.32 cm*	ΔM = 0.74 cm*
		k = 0.34*	k = 0.25*	k = 0.24*	k = 0.2*	k = 0.21*	k = 0.49*

Difference in means and Wilcoxon significance in parentheses on top. K-S test k-value and significance on bottom. A negative ΔM value implies the latter has smaller error. Significance was corrected using Bonferonni’s method with six comparisons. ^†^significant results that did not survive multiple comparisons.

In Table 2, we also show comparisons of error magnitude between blocks. We found significant differences in 63/90 Wilcoxon sign-ranked tests after adjusting for multiple comparisons via Bonferroni’s correction. This table also provides directionality of the error changes, and more participants showed improved hover error than improved touch error after nTNS. Before nTNS, we found the hover condition had higher error in 9 of the 11 significant cases. After nTNS, this was only true for 7 of the 12 significant cases. Fewer cases were present as there was a lower hover condition error in seven participants after nTNS. This was less pronounced in the touch condition, which showed significantly lower error after nTNS in only five participants.

**Figure 4:**
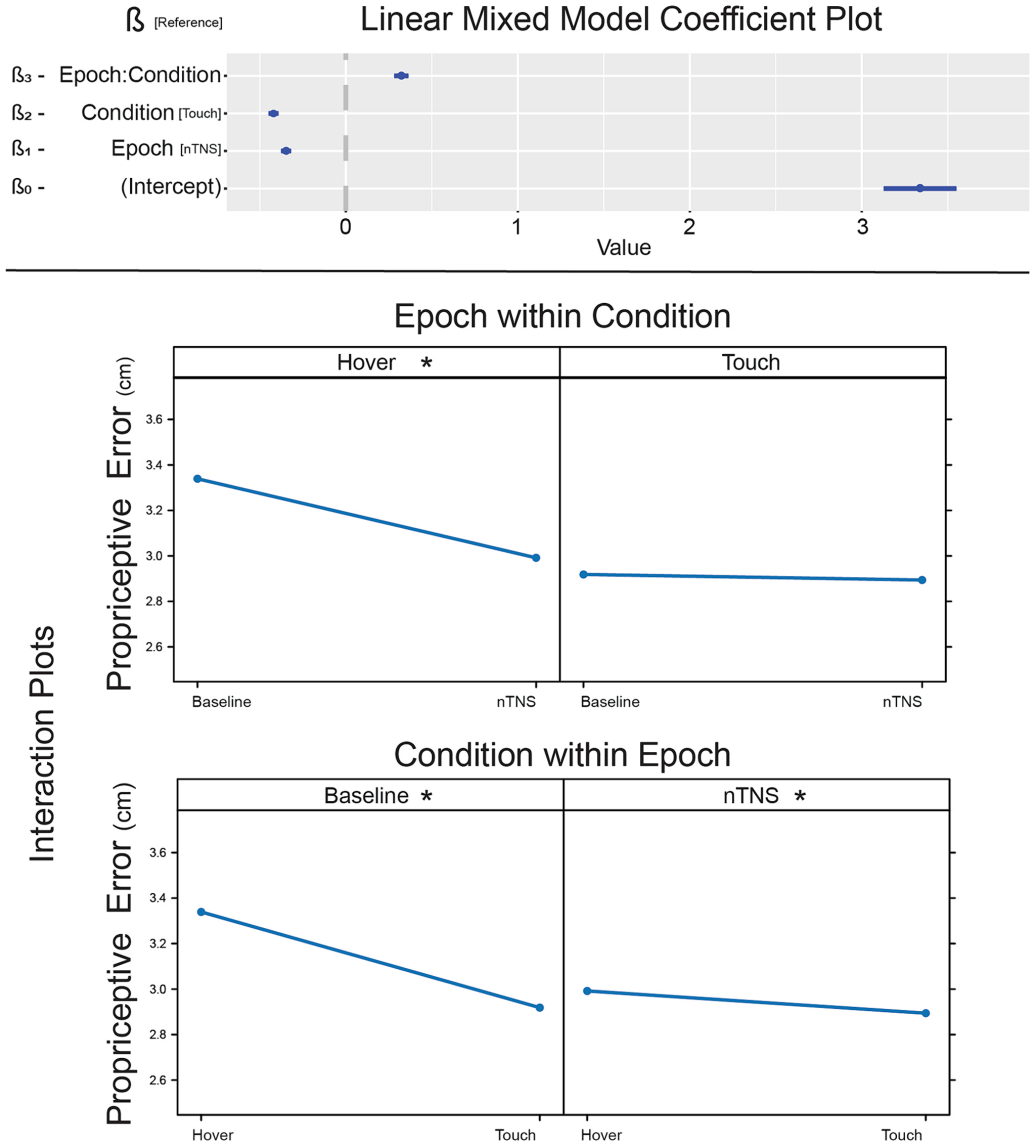
Effect of nTNS on proprioceptive error. [Top] Coefficients of the linear mixed model demonstrating significant factors and interaction effect. [Bottom] Interaction plots of nTNS epochs against touch conditions. *significant *post-hoc* pairwise comparison.

To determine comprehensive results of nTNS effect on error, we implemented a linear mixed-effect model with Condition and Epoch as factors, including the interaction effect. Coefficients and *p*-values for this model are listed in Table 3 and illustrated in Figure 4. Tactile condition (β_2_ = −0.35, *p* < 0.001) and nTNS epoch (β_1_ = −0.42, *p* < 0.001) are significant, suggesting lower error during touch and lower error after nTNS. The interaction term was also significant (β_3_ = 0.323, *p* < 0.001). This overall model supports the conclusion that tactile information and nTNS reduce proprioceptive error, but the relationship is unclear and prompts *post-hoc* analysis.

**Table 3:** Linear mixed model pairwise comparisons.

Linear mixed model			
Coefficient [Reference]	*β*	*t*-value	*p*-value
Intercept (β_0_)	3.39	15.769	<0.001
Epoch [nTNS] (β_1_)	−0.3474	−11.925	<0.001
Condition [Touch] (β_2_)	−0.4207	−14.44	<0.001
Interaction (β_3_)	0.323	7.839	<0.001
Tukey adjustment			
Contrast	Estimate	Z-ratio	*p*-value
Hover [Baseline]—Touch [Baseline] (A)	0.4207	14.44	<0.001
Hover [Baseline]—Hover [nTNS] (B)	0.3474	11.925	<0.001
Hover [Baseline]—Touch [nTNS] (C)	0.4451	15.279	<0.001
Touch [Baseline]—Hover [nTNS] (D)	−0.0733	−2.515	0.0576
Touch [Baseline]—Touch [nTNS] (E)	0.0244	0.838	0.8361
Hover [nTNS]—Touch [nTNS] (F)	0.0977	3.354	0.0044

Tukey’s *post-hoc* pairwise Z ratios and *p*-values for each pair of epoch and condition factor. All main effects are significant.

*Post-hoc* tests were conducted to explore pairwise differences between the two factors: Condition and Epoch (Figure 4). Pairwise comparisons are evaluated employing the Tukey adjustment for multiple comparisons, with estimates and p-values listed in Table 3. The baseline hover block exhibited more error than baseline and modulated touch (Z_A_ = 14.44, *p* < 0.001; Z_B_ = 11.925, *p* < 0.001), which is expected from previous literature (Rincon-Gonzalez et al., 2011; Tanner et al., 2021). After modulation, proprioception without somatosensation (hover) exhibits reduced error (Z_C_ = 15.279, *p* < 0.001). Hover error is reduced to a level no different than unmodulated (Z_D_ = −2.515, *p* = 0.0576) or modulated (Z_E_ = 0.838, *p* = 0.8361) proprioceptive-tactile integration. While the ß-estimate comparing modulated hover against modulated touch is smaller than other significant results, hover error is still significantly higher than touch error after nTNS is administered (Z_F_ = 3.354, *p* = 0.0044), which is congruent with unmodulated hover and touch in this paper literature (Rincon-Gonzalez et al., 2011; Tanner et al., 2021).

## 4 Conclusion

To investigate the effect of non-invasive Trigeminal Nerve Stimulation (nTNS) on tactile-proprioceptive integration, this study observed proprioceptive error in the presence and absence of tactile information before and after nTNS. Using polynomial estimation of end-point estimation error across a workspace, we statistically compared both the error magnitude and the map of proprioceptive error angles. Our primary conclusion is a reduction in pure proprioceptive error with no effect on tactile-proprioceptive integration and no disruption of proprioceptive error maps.

For each of the 15 participants, six pairwise comparisons of error shapes were completed across the four blocks of trials: hover and touch conditions before and after nTNS administration. In all 90 comparisons, K-S tests conclude there were no significant differences in vector magnitude distributions. Confirming previous literature, the proprioceptive error maps were stable across blocks (Tanner et al., 2021; Rincon-Gonzalez et al., 2012). We can contribute the novel conclusion that nTNS does not change error maps and they remain stable after modulation. As nTNS and other cranial nerve stimulation modalities are utilized in neuromuscular or motor rehabilitation (Cook et al., 2020; Fallahi et al., 2023; Gao et al., 2023), this offers assurance that neuromodulation will not acutely confound existing movement or estimation strategies.

In our linear mixed effect model, we see a general decrease in the error when tactile information is integrated, with significant pairwise comparisons between each Hover and Touch pair. Integrating tactile feedback always produces less error than pure proprioception. This relationship is present before nTNS within the baseline epoch, which confirms previous literature. After nTNS, the same tactile-proprioceptive integration reduction in error is present, but to a smaller degree. The other pairwise comparisons offer interesting insights to the nTNS effects. Pure proprioception after nTNS (nTNS Hover) is not significantly different than using tactile feedback before nTNS (baseline Touch), suggesting error estimation improved unrelated to improved tactile-proprioceptive integration. This could imply heightened attention to the task in general or a better ability to interpret the proprioceptive information alone.

The lack of significant difference between the touch condition in both epochs confirms the lack of nTNS effect on tactile-proprioceptive integration. The similarity between baseline touch and nTNS hover is striking, as it suggests modulated proprioception is comparable to default tactile-proprioceptive integration. Without defining the mechanisms, we can conclude that acute nTNS allows a single channel of proprioceptive information to be comparable to bimodal information in a task requiring a cognitive response. Sensory perception May be a function of attention which can be modulated by nTNS, while tactile-proprioceptive integration is related to previous sensory experience and sensory familiarity (Tanner et al., 2021). As tactile-proprioceptive integration is unaffected by acute neuromodulation in this study, error is likely modified by attention and not integration. It is unknown if this would be consistent in a task with accelerated responses that requires higher attentional demand, such as a race-model reaction time task with a single versus bimodal stimuli. Accelerated tasks indirectly model tactile-proprioceptive neural summation independent of cognitive task (Forster et al., 2002; Zehetleitner et al., 2015), and would efficiently probe sub-perceptual integration.

Alternatively, the results suggest the possibility that the difference between hover and touch May reflect a process separate from tactile-proprioceptive integration: somatosensory LC activation. Our working hypothesis of the nTNS effect is that it activates attention and arousal via synaptic input from the trigeminal nucleus to the LC through direct synaptic inputs from the nucleus of the solitary tract (NTS; Schwarz and Luo, 2015; Zerari-Mailly et al., 2005; Contreras et al., 1982), but also transit through the nucleus paragigantocellularis, prepositus hypoglossi, and the reticular formation (de Cicco et al., 2018). Tactile input activates LC at short latencies, exhibiting enhanced firing rates like arousal states (Foote et al., 1980). This raises the possibility that lower error in baseline touch conditions is due to increased arousal. Without nTNS, this would be due to heightened LC activity from natural tactile input (Rincon-Gonzalez et al., 2011). In our nTNS epoch conditions, we suggest nTNS induces LC arousal and both hover and touch conditions demonstrate decreased, but comparable, error. In short, nTNS induces an arousal state that tactile inputs normally provide, causing hover trials to exhibit similar error to touch trials.

While the comprehensive analysis demonstrates compelling results, Table 2 illustrates some variability in the individual response to nTNS. Specifically, participants JA and NB demonstrate a poor response to the nTNS, either with significantly higher error or no significant difference in epochs. However, both participants also demonstrate significantly higher error in the baseline epoch touch condition versus the hover condition. This is contradictory with typical tactile-proprioceptive integration and could indicate poor task comprehension or poor default tactile-proprioceptive integration. Regardless of these cases, it is clear not all subjects demonstrate a reduction in proprioceptive error after receiving nTNS. From literature, we know there is variability in individual response to neuromodulation, specifically for motor rehabilitation. This variability has been linked to specific biomarkers such as genetic polymorphisms (Cheeran et al., 2008; Antal et al., 2010; Fritsch et al., 2010). This study is not powered to evaluate genetics, nor is it in the scope of the resources. However, such sources of variability are necessary to consider in effective larger studies.

## Data Availability

The original contributions presented in the study are publicly available. This data can be found here: https://osf.io/2mzng/?view_only=3cbc95a17a144de8b47b2d801c73ce4e.
